# Flapless Cone Beam Computed Tomography-Guided Implant Surgery with Contextual Transcrestal Sinus Lift Augmentation Using New Bone Compactor Tools

**DOI:** 10.1155/2020/8873234

**Published:** 2020-12-07

**Authors:** Ferdinando Attanasio, Sergio Bortolini, Daniele Carbone, Andrea Pacifici

**Affiliations:** ^1^Department of Health Sciences, Magna Graecia, University of Catanzaro, Catanzaro, Italy; ^2^CHIMOMO, Università di Modena e Reggio Emilia, Largo del Pozzo 71, 41125 Modena, Italy; ^3^Department of Oral and Maxillo-Facial Science, Sapienza University of Rome, Rome 00100, Italy

## Abstract

In the present paper, the authors present a case report of premolar edentulism in the upper jaw treated through a guided flapless oral implant surgery with contextual crestal sinus lift, performed with a system of manual screw-tapered bone expanders (B&B Dental, San Benedetto, BO, Italy). The surgery was planned by means of dedicated software, through which the data obtained from the CBCT and from intraoral scanner impression were matched, with consequent production of a surgical template. The proposed surgical procedure is minimally invasive, very simple, and fast and ensures good comfort for the patient by avoiding the elevation of mucoperiosteal flaps and uncomfortable malleting maneuvers. In addition, the presented method shows a good degree of correspondence between the ideal position of the implant in the planning phase and the actual one detectable after the surgery.

## 1. Introduction

A tooth lost in the posterior areas of the upper jaws besides leaving a chewing deficit can also lead to a slow but inexorable expansion of the maxillary sinus in the side affected by edentulism [1, 2]. The pneumatization of the maxillary sinus therefore brings about a lessening of the available surgical space for the implant insertion without recurring to any bone augmentation procedure [3]. International reports in various studies [4, 5] have underlined the anatomical limits that allow the surgeon to obtain a better prognosis for the prosthetic rehabilitation on implants in posterior edentulous areas of the upper jaw [6]. In particular, a residual ridge lesser than 5 mm in height requires a surgical approach through the elevation of a lateral window, while a residual ridge greater than 5 mm in height can be approached through a transcrestal elevation procedure with excellent success rates [7]. Several different techniques have been proposed by various authors for transcrestal sinus elevation surgery: from the osteotomy proposed by Summers [8, 9] to the more recent fluid dynamic technique [10] or the use of a compactor/expander or even the use of dedicated implants which allow the insertion of the fixture and the contextual elevation of the sinus membrane [11–13].

In this case report, the authors propose a computer-guided surgery procedure consisting in crestal maxillary sinus lift performed with bone expanders (B&B Dental, San Benedetto, BO, Italy) and contextual oral implant insertion. Through the exposed technique, instead of penetrating the bone tissue with osteotomes by percussion in the apical direction (for example, the Summers type), we have the insertion of a series of compactors with a gradually increasing diameter, which allow the safe fracture of the maxillary sinus floor resulting in a totally atraumatic and painless procedure for the patient. This protocol allows raising the height of the ridge by even 3-4 mm in the antral direction. Moreover, the preparation of the implant site through the use of the compactors allows increasing the density of the bone in the implant site in order to obtain higher insertion torque and gain a greater primary stability of the fixture compared to traditional implant site preparation drills or to percussion osteotomies of Summers [14]. In order to obtain even greater bone volume around the apical zone of the implant, it is possible to insert an autogenous bone graft or bone substitute [15]. This maneuver must be performed with accuracy and precision in order to avoid the laceration of the Schneiderian membrane [16].

## 2. Case Presentation

**Figure 1:**
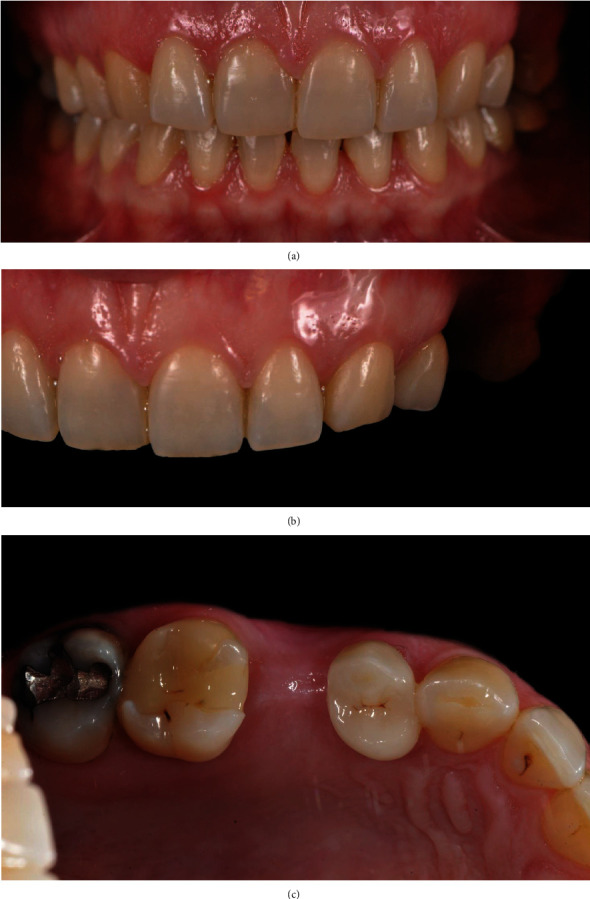
(a) Intraoral preoperatory frontal view. (b) Intraoral preoperatory frontal view. (c) Intraoral preoperatory occlusal view.

A healthy 34-year-old woman presented to our observation requesting clinical evaluation of her upper left maxilla and implant prosthetic treatment of second left upper premolar edentulism (Figures 1(a)–1(c)). The missing tooth was extracted several years before due to a history of dentoalveolar infections. The patient reported no smoking or significant illness while referring good oral hygiene habits.

**Figure 2:**
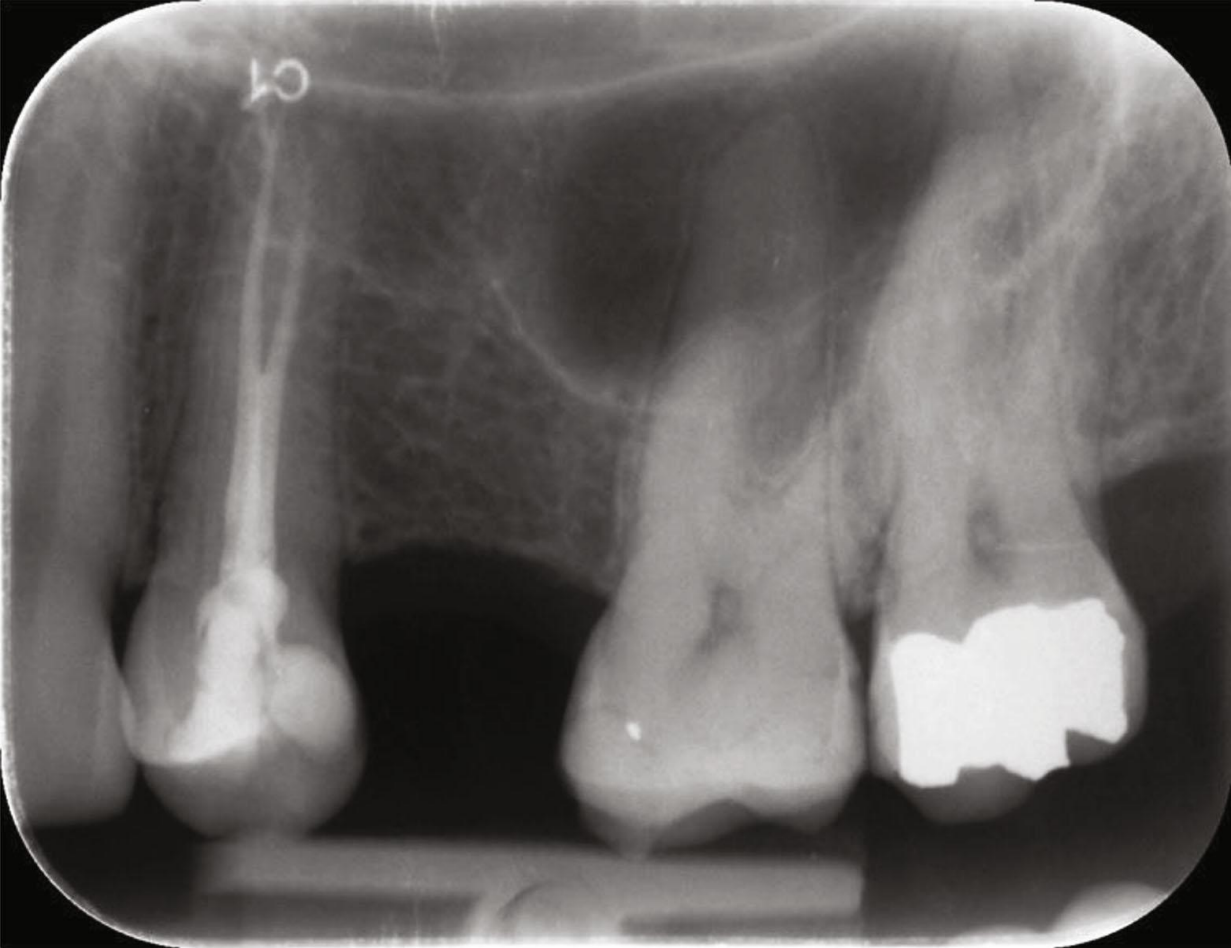
Preoperatory endoral radiography.

As a predictable, significative vertical ridge defect was present, probably due to both bone resorption and maxillary sinus expansion, which made the anatomical site seriously compromised and inadequate for implant-supported rehabilitation. In fact, the radiographic examination clearly displayed that the bone height in this area was not sufficient for standard length implant placement (5 mm bone height) while the horizontal bone thickness appeared to be sufficient (Figure 2).

The treatment plan included the immediate placement of one fixture with a contextual transcrestal sinus lift procedure to ideally restore the shape, function, and esthetics of the alveolar defect. In order to minimize the trauma as much as possible, avoiding flaps from opening and increasing the bone volume only for the necessary portion, we decided to perform the preparation of the implant site and the positioning of the implant through static computer-aided implant surgery.

In order to proceed with the computer-assisted implant surgery, a CBCT (Pax i-3d Green, Vatech, Yongin, Korea) of the affected area was preliminarily performed. Furthermore, a digital acquisition of the dental arch's surface profile was performed through an intraoral scanner (CEREC Omnicam v4.5.1; Dentsply Sirona), and the obtained .stl file has been extracted. The two files have been imported into dedicated software (B&B Dental Guide System, B&B Dental San Benedetto, BO, Italy) so as to allow the digital superimposition of the .stl file containing all the data of soft tissue and teeth and the .dcm file that brings along the bone and internal anatomical structures' information in order to allow the correct digital positioning of the proper size and kind implant for the structure of the upper jaw. The .dcm and .stl file matching was performed to overlap the obtained information and clearly display the patient's situation in a very detailed manner. By the idea that the patient is semiedentulous, the acquisition of the CBCT was made without the use a radiographic template with radiopaque markers, as the B&B Dental Guide System software allows the overlapping of the impression made by Omnicam v4.5.1 (Dentsply Sirona) directly with the .dicom file by recognizing some anatomical portions of the residual teeth.

Surgeons evaluated the obtainable volumes through the sinus lifting procedure and then digitally positioned an implant of 4∅×8 mm length (Duravit 3P, B&B Dental, San Benedetto, BO, Italy), suitable with the anatomical structure and in a prosthetically guided positioning to obtain screw-retained rehabilitation.

**Figure 3:**
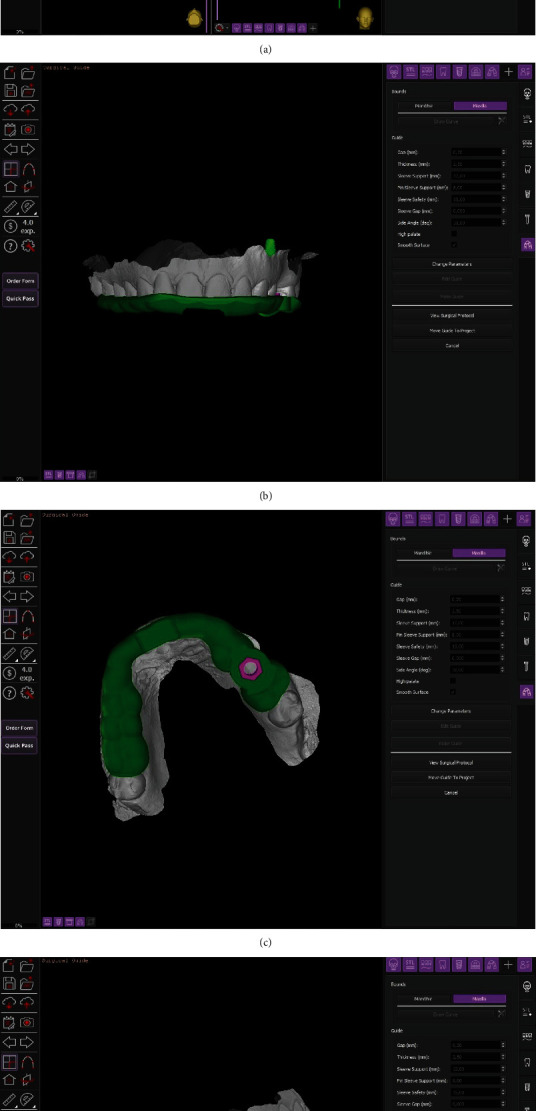
(a) Software evaluation and digital planning of the surgical procedure. (b–d) Digital modeling of the surgical template.

In order to precisely determine the depth of the osteotomy, it is necessary to use cross-sectional images of the elevation site and measure the distance between the top of the bony crest and the maxillary sinus floor along with the implant axis on the three-dimensional model (Figures 3(a)–3(d)).

As a result of the evaluations made through the software, it was possible to perform an accurate digital implant positioning and to design a template and export the resulting .stl file. This file can be sent to any laboratory for printing; in our case, we relied on B&B Dental guided surgery and printing service to receive both a printed model and the finished surgical template.

The surgical template is printed in acrylic material and contains hexagonal polyether ether ketone (PEEK) sleeves (4.2∅×5 mm height) in correspondence with the planned implants' position and with their own inclination. The internal diameter of the sleeves perfectly fit the neck of all the instruments inside the dedicated guided surgery kit (B&B Dental guided surgery kit, B&B Dental, San Benedetto, BO, Italy) in order to accurately direct the instrument to prepare the planned osteotomy. All the drills' necks are 9 mm long while the cutting edges have variable sizes according to the producer's implant sizes.

The chosen material for the sleeves allows lower dimensional tolerance, thus improving the accuracy of the sleeve instrument fitting in order to guarantee higher precision in terms of directing the drill while avoiding any temperature rising.

**Figure 4:**
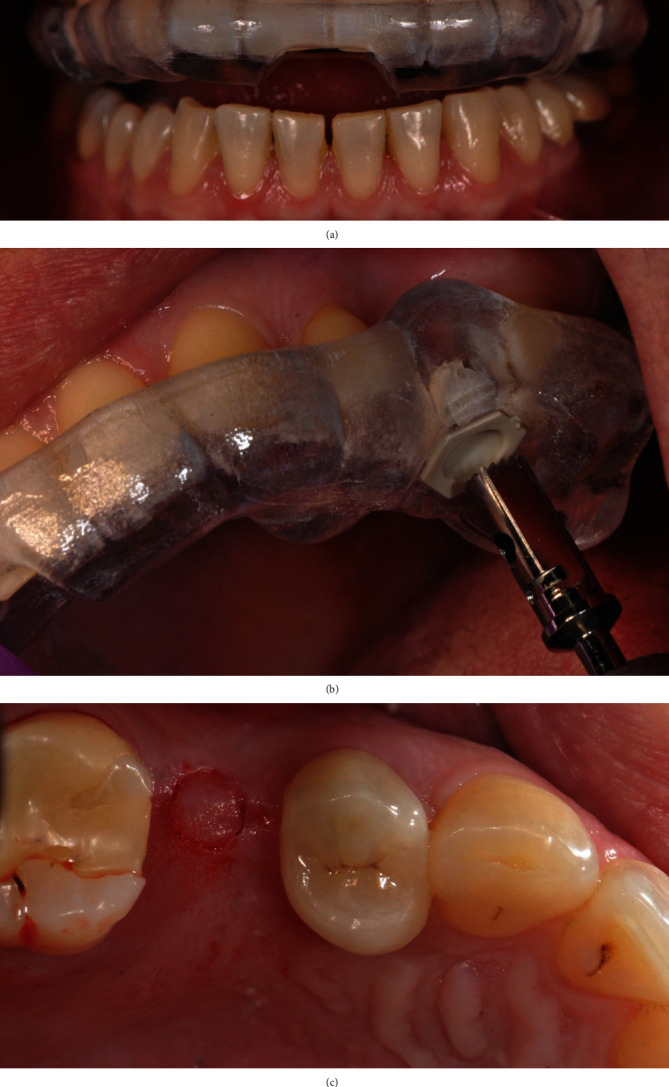
(a) Template positioning before the surgery. (b) Rotating circular tissue punch. (c) Operculectomy through the gingiva.

Before starting the procedure, the surgical template is checked for proper seating and secured in place with a tooth support (Figure 4(a)), thus ensuring the greatest possible precision [17, 18].

The stabilization of the surgical template is a key point to reproduce the virtual surgery in the mouth of the patient with high accuracy [19]. The loss of accuracy may result from movements of the surgical guide during implant preparation or from the so-called “intrinsic” error of the template [20].

The patient was premedicated with amoxicillin+clavulanic acid (2 g) 1 hour before surgery, and then, 875 mg of amoxicillin and 125 mg of clavulanic acid were administered twice per day for 1 week following the surgery.

The patient rinsed with a 0.20% chlorhexidine gluconate solution (Curasept, Curaden HealthCare, Italy) for 1 minute; then, the skin surrounding the surgical site was disinfected. The patient presented a thick biotype. Under local anesthesia (2% lidocaine with 1 : 80,000 epinephrine), an operculectomy was performed with the use of a rotating circular tissue punch mounted on a handpiece, after which a pilot drill was used to cut the cortical bone and assess the tissue consistency (Figures 4(b) and 4(c)).

**Figure 5:**
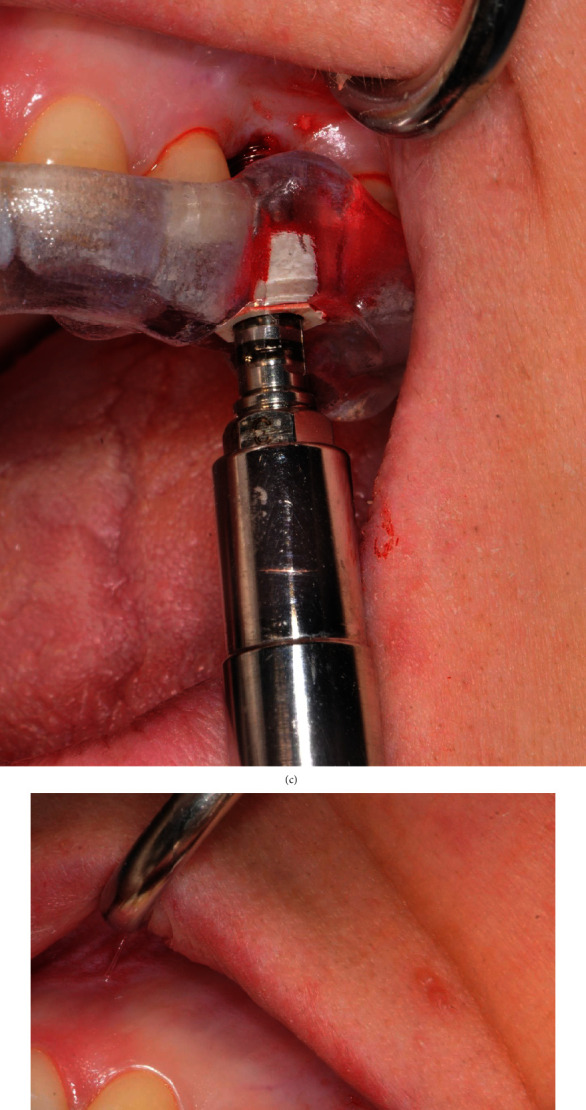
(a–c) Osteotomy by manual bone compactors (B&B Dental guided surgery kit, B&B Dental, San Benedetto, BO, Italy). (d) Filling of the implant site with inorganic bovine bone-derived mineral. (e) Dental implant positioning. (f) Positioning of the prosthetic abutment.

Subsequently, bone compactors with a convex apex of increasing diameter and length were inserted with a clockwise rotation. We started with a 2.2 mm in diameter compactor for a working length of 6 mm (so as to reach the floor of the maxillary sinus); then, the following compactors were 3.0, 3.5, and 4.0 mm in diameter and have been used in sequence up to a length of 10 mm (Figures 5(a)–5(c)).

Once the osteotomy for the preparation of the implant site was finished, the patient performed the Valsalva maneuver, which showed the absence of perforations of the sinus membrane. The implant site was filled with inorganic bovine bone-derived mineral (ABBM) (Bio-Oss, Geistlich Pharma, Wolhusen, Switzerland) (Figure 5(d)).

The convex rounded tip of the compactors allows performing compressions and expansions of the alveolar bone by pushing and compacting it at the ends of the implant site (both vertically and horizontally). This allows not only fracturing the floor of the maxillary sinus completely and atraumatically but also, above all, lifting the Schneiderian membrane without the need to use other instruments.

At this point, using the mounter from the surgical kit, a soft thread 4∅×8 mm long implant was placed (Figure 5(e)). The mounter has the function of guiding the final insertion of the implant in terms of angle, height, and orientation. This component has a landmark hexagon whose faces are aligned with those of the implant's hexagonal connections. The faces of the hexagon must be matched to the direction of the sleeve inside the surgical guide that has been planned to allow the correct positioning of the prosthetic abutment (Figure 5(f)).

**Figure 6:**
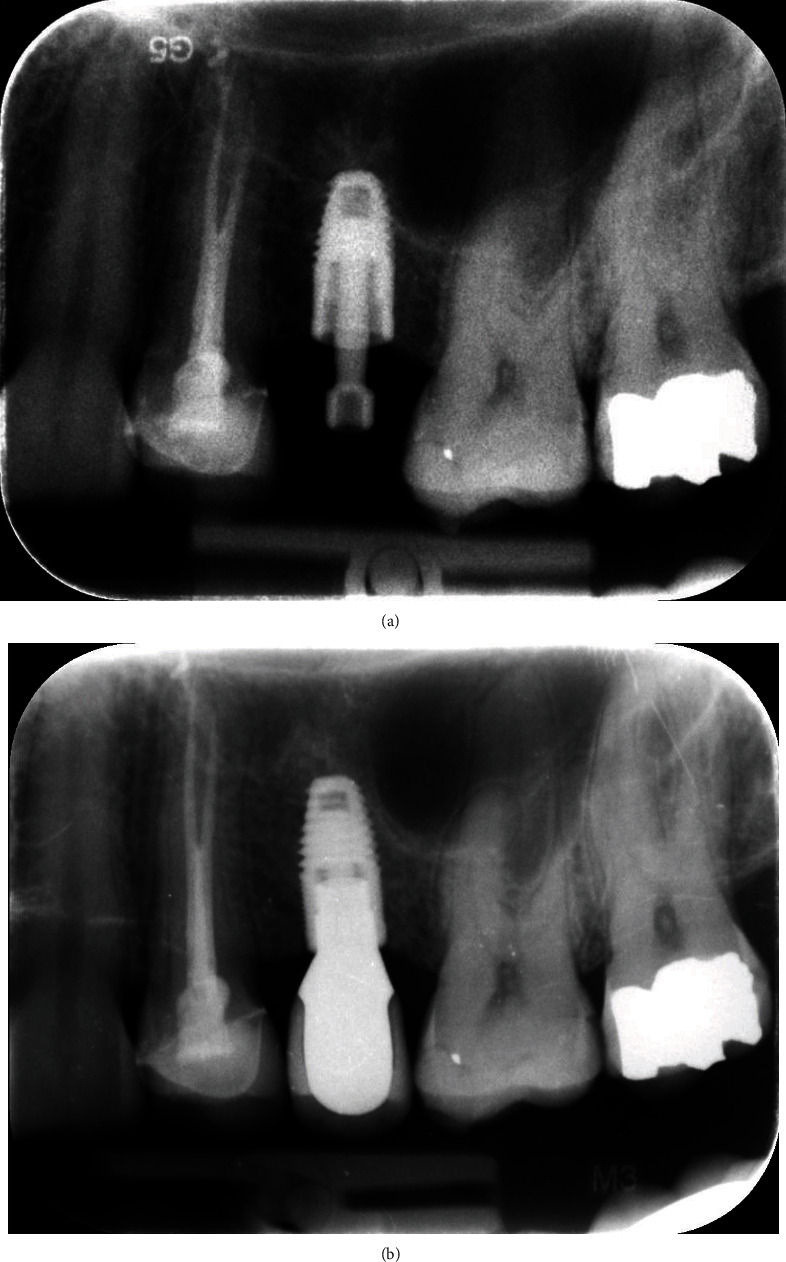
(a) Postoperatory endoral radiography. (b) Endoral radiography taken after 9 months from the surgery.

The precision of the final implant position was monitored through an endoral X-ray (Figure 6(a)).

Before the end of the surgical procedures, an optical impression with CEREC Omnicam was taken and a comparison between the project and the clinical outcome was made through a digital superimposition. This showed that, despite the contextual positioning of the implant and the transcrestal sinus elevation, there were 1.88 mm of deviation on the position of the apex, 0.96 mm of deviation at the head of the implant, and an angle deviation of 4.73°.

At the end of the surgery, a transmucosal healing screw was placed and no sutures had to be inserted.

In addition to the pre- and postoperative antibiotics described above, an anti-inflammatory medication (ibuprofen 600 mg) has been administered immediately after the surgery and was thereafter prescribed three times a day for 1 week following surgery. 0.20% chlorhexidine digluconate mouthwash rinses were prescribed twice daily for ten days, starting 24 hours after the surgery. It was not possible to carry out radiographic control 4.5 months after the surgery due to the patient's pregnancy. Before proceeding with the impressions for the prosthetic restoration, resonance frequency analysis (Osstell ISQ™ device, Integration Diagnostics AB, Göteborg, Sweden) of the osteointegrated implant was performed: values of 81 in the B/L direction and 80 in the M/D were recorded.

About 5 months after the surgery, a screw-retained implant-supported metal porcelain crown was applied; all the laboratory steps followed a completely digital workflow. Intraoral examination showed healthy a peri-implant mucosa without suppuration, swelling, or erythema close to the implant site. The patient did not complain of foreign body sensation, pain, or dysesthesia.

Endoral radiographies performed immediately after the end of the procedure and after 9 months (4 months after the implant underwent prosthetic loading) showed good osseointegration of the fixture and the absence of pathological signs (Figure 6(b)).

## 3. Discussion

The rehabilitation of partial or total edentulous patients using implant prosthesis techniques has become a routine practice with reliable long-term success rates [21, 22]; however, local conditions of the alveolar edentulous ridge can seriously disadvantage implant positioning. In particular, the posterior edentulous jaw bone has often represented a challenge for oral surgeons because of the insufficient bone volume, as a consequence of crestal resorption caused by the atrophy of the alveolar process and the expansion of the maxillary sinus. Moreover, the quality of the residual bone can further reduce the primary stability of implants [23].

Procedures for sinus floor elevation have become an argument of great interest since the introduction of the technique by Tatum in 1976 [24, 25]. The first publication dates back to 1980, in an article by Boyne [26].

The traditional technique for the maxillary sinus floor augmentation foresees the opening of a lateral window, the elevation and medialization of the membrane with rounded off instruments, the grafting of particulate material, and the use of a membrane to cover the access window.

Historically, the maxillary sinus lift surgery that was performed in the early 80s involved hospitalization of the patient. During the procedure, an autologous bone graft was used in block or in the form of particulates, with simultaneous or deferred placement of endoosseous implants [26–28]. This technique was also widely used in the 90s [29–31]. The autologous block grafts within the preimplant regenerative oral surgery showed several critical issues including reduced survival rate and postoperative discomforts [27].

To get around these problems, researchers studied the use of heterologous bone graft substitutes [32, 33]. Moreover, research into the guided bone regeneration brought about the introduction of membranes to cover the lateral access window [34, 35].

The technique of maxillary sinus floor elevation and bone grafting through lateral window osteotomy is commonly performed, nowadays, with foreseeable results in an outpatient setting, avoiding in this way the hospitalization of the patient; moreover, the choice between immediate or deferred implant insertions depends on the capacity of the surgeon to obtain a good primary stability [36, 37]. According to the revision of Wallace and Froum [38], once the primary stability has been reached, the difference in the survival rates between the implants positioned contextually or after the graft is unimportant.

The maxillary sinus floor elevation technique through a lateral access window is usually used when the height of the residual bone is inferior to 4-5 mm.

In 1994, Nedir et al. [7] introduced a sinus floor augmentation technique through a transcrestal access; from that moment, a number of modifications to Summers' technique have been introduced [9–43].

Exploiting the teachings from techniques proposed in literature, the “Duravit Crestal Sinus Lift” systematics of the B&B Dental Implant Company presents some modifications which permit, with minimal surgical invasion, the obtainment of good results in cases of maxillary bone atrophy with residual bone of at least 4 mm in height. Moreover, the use of a manual technique permits a notable increase in the sensibility of the surgeon during the operation; avoiding the use of a hammer for bone compaction also makes the surgery less traumatic for the patient.

The simplicity of the use of this method also allows the less expert surgeon to speed up the learning curve and to safely position an implant of standard dimensions in areas with important atrophy in an outpatient setting.

The application of digital technologies in the various branches of dentistry represents another topic that is gaining increasing interest in literature. In the field of oral implantology, digital technologies find various possible uses, including optimizing the planning of the therapeutic program as well as developing a computer-guided surgical procedure [44].

The introduction of technologies such as intraoral optical scanners and CBCT in dental practice has made possible to develop diagnostic and planning protocols through the digital processing of anatomical data by means of virtual models, which represent a precise representation of anatomy of the patient [44]. Among the several possible applications, we have the possibility of planning the proper dental implant position and obtain surgical templates through the digital matching of the anatomical data obtained from the optical scans of the intraoral surface with those obtained from the CBCT [45]. According to the evidence in literature, indications for guided implant surgery could be the need for minimally traumatic or flapless surgery, optimal implant positioning, and immediate loading [46].

To date, several authors have studied these procedures using different systems and measuring the deviation between the planned position for the implant and the actual one after its insertion. In particular, the parameters most frequently used to measure the degree of precision of given systematics are the deviation of the implant position in its most coronal and apical portions, as well as its angulation [18, 47–49]. In the present report, a dental implant was positioned contextually to transcrestal sinus elevation, resulting in 1.88 mm of deviation on the position of the apex, 0.96 mm of deviation at the head of the implant, and an angle deviation of 4.73°. However, these data are in line with those documented in literature [18, 47–49].

In conclusion, correct treatment planning is fundamental for prosthetic-guided oral implantology and modern technologies can help to position dental implants in the most suitable location. Furthermore, the development of increasingly less invasive surgical procedures represents a topic of crucial importance in modern medicine as it allows, among other things, reducing discomfort for patients and treatment times.

The systematics presented by the authors in this case report allows the simultaneous insertion of dental implants with simultaneous elevation of the maxillary sinus floor with a transcrestal approach, without performing access flaps or invasive maneuvers such as hammering of osteotomes. The minimally invasive surgical procedure is digitally planned and guided through a custom-made surgical template.
